# Synergistic Effect of Screen-Printed Single-Walled Carbon Nanotubes and Phosphorylated Cellulose Nanofibrils on Thermophysiological Comfort, Thermal/UV Resistance, Mechanical and Electroconductive Properties of Flame-Retardant Fabric

**DOI:** 10.3390/ma14237238

**Published:** 2021-11-26

**Authors:** Tjaša Kolar, Vanja Kokol

**Affiliations:** Faculty of Mechanical Engineering, University of Maribor, Smetanova Ulica 17, 2000 Maribor, Slovenia; tjasa.kolar2@um.si

**Keywords:** single-walled carbon nanotubes, phosphorylated cellulose nanofibrils, screen-printed fabric, thermophysiological comfort, thermal and UV resistance, electroconductivity

## Abstract

Single-walled carbon nanotubes (SWCNTs) and phosphorylated nanocellulose fibrils (PCNFs) were used as functional screen-print coatings on flame-retardant (FR) fabric, to improve its thermal resistance and thermophysiological comfort (wetting, water vapour and heat transmission) properties, while inducing it with electrical conductivity and UV protection. The effect of PCNF printing, followed by applying a hydrophobic polyacrylate (AP), on the same (back/B, turned outwards) or other (front/F, turned towards skin) side of the fabric, with and without the addition of 0.1–0.4 wt% SWCNTs, was studied by determining the amount of applied coating and its distribution (microscopic imaging), and measuring the fabric’s colour, air permeability, thickness, mechanical, flame and abrasion resistance properties. Due to the synergistic effect of PCNF and SWCNTs, both-sided printed fabric (front-side printed with PCNF and back-side with SWCNTs within AP) resulted in an increased heat transfer (25%) and an improved thermal resistance (shift of degradation temperature by up to 18 °C towards a higher value) and UV protection (UPF of 109) without changing the colour of the fabric. Such treatment also affected the moisture management properties with an increased water-vapour transfer (17%), reduced water uptake (39%) and asymmetric wettability due to the hydrophilic front (Contact Angle 46°) and hydrophobic back (129°) side. The increased tensile (16%) and tear (39%) strengths were also assessed in the warp direction, without worsening the abrasion resistance of the front-side. A pressure-sensing electrical conductivity (up to 4.9∙10^−4^ S/cm with an increase to 12.0∙10^−4^ S/cm at 2 bars) of the SWCNT-printed side ranks the fabric among the antistatic, electrostatic discharge (ESD) or electromagnetic interference (EMI) shielding protectives.

## 1. Introduction

Resistance to heat and fire from the environment towards the human body are the main functional properties of flame-retardant (FR) textiles, which is achieved through thermal stability and insulating ability. In addition, such textiles must also dissipate metabolic heat and body vapours away from the body, thus ensuring good physiological comfort during wearing when exposed to such an environment [1].

A nanotechnology supported FR-protective coating containing a wide range of materials, such as metal and carbon based nanoparticles (graphite, carbon black, carbon nanotubes (CNTs) [2]) with up to 10% of loading, applied as single or multi-layer coatings with thicknesses up to a few tens µm [3], are thus proposed to impart such multifunctional properties without affecting the original material properties (such as flexibility, rigidity and stiffness). Such coatings act as thermal insulators, absorbing the heat and oxygen from the atmosphere and blocking their transfer through the textile. In addition, they can induce textile materials with UV, antistatic and electromagnetic/EMI shielding protections, or even electrical conductivity [4], the properties whose demand has been growing enormously in recent years [5] due to the wide range of forthcoming conventional and innovative applications, ranging from apparel and sportswear [6], sensing devices [7], wearable electronics [4] up to flexible heating equipment [8]. CNTs seem to be the most appropriate in this respect due to their own remarkable electrical conductivity (10^6^–10^7^ S/m [9]), exceptional thermal conductivity (3500 W/Mk [10]), high UV adsorption [11], high tensile strength (30–45 GPa) and yield strain (5.3–5.8%) [12], light weight, low density and flexibility [13]. 

Textiles have been functionalised with CNTs by several approaches, like weaving or knitting of carbon-based fibres and yarns into textiles, by electrodeposition, spinning or various coating techniques [4,13,14], where dipping [13,14,15,16,17] or screen-printing [18] were used as one of the simplest and most commonly used ways. Since CNTs are prone to agglomeration and the uniform distribution of CNTs is the most crucial factor rendering the final properties of the product, CNTs were used in combination with citric acid [14,18], sodium hypophosphite [18], aliphatic urethane acrylate [5], polypropylene [19,20], 3-glycidyloxypropyl-trimethoxysilane, p-phenylenediamine and methanol [13], poly(ethylenimine), ammonium polyphosphate [21], butyl acrylate [16], aromatic azide polymer [17], etc., to obtain homogeneous dispersibility, as well as good crosslinking with the textile material. Thus functionalized textiles also assist in heat dissipation [10], while also having super-hydrophobic properties (contact angle above 150°) [16,18,22], good UV protection (ultraviolet protective factor/UPF above 50 [11,22]), and improved mechanical [13,15] properties. In addition, CNTs treatments using concentrations from 0.015 wt% [23] up to 8 wt% [19] resulted in fabrics with conductivities ranking from 2∙10^−3^ S/cm [18] up to 2.7 S/cm [24], or sheet/square resistances in the range of 10^2^ Ω/sq [13]–8.3∙10^11^ Ω/sq [20]. 

In our previous study, we showed that screen-printed microfibrillated cellulose (MFC) on flame-retardant (FR) fabric increased its moisture build-up significantly without compromising its water vapour transfer [25], thus, improving the thermophysiological comfort properties of hydrophobically post-coated fabric. 

In this work, more thermally stable [26] phosphorylated nanocellulose (PCNF) with even better moisture absorption ability [27] and good colloidal dispersibility due to the presence of anionic phosphorous acid groups on the cellulose fibrils is used, in combination with single-wall CNTs (SWCNTs) to improve the fabric’s thermophysiological comfort, flame retardancy and UV protection, while simultaneously obtaining an electro-conductive surface with an antistatic, electrostatic discharge or electromagnetic shielding protection properties. To achieve this goal, the fabric was first printed with PCNF (on its front or back side) and then with a hydrophobic polyacrylate (AP), applied on the fabric back-side, with or without different content of SWCNTs pre-dispersed in the water-based acrylic system. The influence of the coating’s mass on the fabric’s thickness, air permeability, surface wetting, thermal and water-vapour resistance were considered, followed by assessing of its colour change, UV protection, electrical conductivity, mechanical, flame and abrasion resistance properties. 

## 2. Experimental

### 2.1. Materials

The two-wefts woven flame-retardant (FR) fabric of 145 ± 1.4 g/m^2^ mass (ISO 3801 [28], 0.332 ± 0.02 mm thickness (ISO 5084:1996 [29]), and 36 threads/cm in the warp direction and 51 threads/cm in the weft direction (EN 1049-2 [30]), made from a mixture of meta aramid and FR Lenzing viscose (34/34% ratio) predominant in the warp direction and on the back-side (i.e., the side facing outwards during wearing), and viscose filaments (32%) predominant in the weft direction and on the front-side (i.e., the side facing the wearer), was provided by Tekstina Ltd., Ajdovščina, Slovenia.

Water suspended phosphorylated nanofibrillated cellulose (PCNF) of 2–3 μm long and highly branched 10–200 nm wide fibrils with around 0.13 degrees of substitution and zeta potential of around −45 mV at pH 10, as determined by conductometric titration, were provided by Xylocel Oy, Espoo, Finland.

Commercially available (Tuball Matrix 302) 10 wt% concentrated SWCNTs paste (well-dispersed in about 70 wt% of distyryl biphenyl derivative, specific substances covered by NDA) with dimensions of 1.6 ± 0.4 nm in diameter, length of >5 μm, a length/diameter ratio of 5000, a specific surface area of around 1000 m^2^/g, 10^3^ S/cm of electrical and 6600 W/mK of thermal conductivity, was purchased from OCSiAl, Luxembourg. 

The acrylate paste (AP, pH of 8–8.5) was prepared by Tekstina Ltd., Slovenia, consisting of 133.3 g/kg polyacrylate self-crosslinking binder (Tubifast AS 30), 1.67 g/kg ammonium water, 2.5 g/kg polymeric silicone as an antifoam (CHT entschaumer BSN), 20 g/kg melamine resin (Tubigat WAF 20), 18 g/kg ammonium salt of polymeric carboxylic acids as a thickener (Tubivis DRL 600), acrylic-acid as a rheology additive (Tubigat R 130), all commercial products of CHT Bezema, Germany.

### 2.2. Preparation of PCNF-Based Dispersions and Acrylate-Based Pastes

The viscosity of both the pastes and dispersions was evaluated using a Haake rotational Viscotester V2 (Thermo Fisher Scientific Inc., Waltham, MA, USA) at room temperature. 

The 1.5 wt% water-dispersed PCNF (viscosity of 45 ± 2 dPas) was used throughout the study. The PCNF dispersions were also prepared with the addition of different content (0.1, 0.2 and 0.4 wt%) of SWCNTs, or 2.9 wt% of SWCNTs-dispersing agent (Disp; corresponding to the addition of 0.4 wt% SWCNTs) by mixing them for 15 min with a rotary mixer (RE 166, IKA-Werke, Staufen, Germany), resulting in a viscosity of 80 ± 2 dPas, 90 ± 2 dPas, and 120 ± 2 dPas or 40 ± 2 dPas, respectively. 

The viscosity of the acrylate paste (AP) was adjusted to 105 ± 5 dPas with the addition of distilled water. APs of the same viscosities were also prepared with different content (0.1, 0.2 and 0.4 wt%) of SWCNTs, or 2.9 wt% of SWCNTs-dispersing agent (Disp) by mixing them for 15 min with a rotary mixer.

Both PCNF dispersions and AP pastes were designated according to the amount of SWCNTs added, where the number (0.1, 0.2 or 0.4) denotes the different concentrations of SWCNTs (0.1, 0.2 and 0.4 wt%, respectively). The dispersions/pastes containing only SWCNTs-dispersing agent were designed as Disp.

### 2.3. Screen-Printing Process

The printing was performed on a Zimmer laboratory screen-printing machine using a nickel-based rotary screen (SPGPrints B.V., Boxmeer, The Netherlands, formerly Stork) of 60 mesh size (14% open area, 161 μm holes’ diameter, 100 μm screen plate thickness) and a steel-rod type squeegee (diameter 15 mm) at magnet-adjustable pressure (no. 6) and manually-set speed (level 5 ≈ 6 m/min). 

The fabric was printed on the same or different sides in two-printing steps. In one-sided printing, the PCNF based dispersion (with or without SWCNTs) was first applied on the back-side (facing outwards during wearing) followed by AP, while for both-sided printing, the PCNF (with or without SWCNTs) was applied first on the front-side (facing towards the wearer), and then AP (with or without SWCNTs) on the fabric’s back-side. In addition, the printings of only AP and AP containing Disp were performed, acting as the references, together with the non-treated fabric. After each printing step, the samples were dried at 100 °C for 3 min, and additionally exposed to 170 °C for 2 min, in order to fix the AP coating by using a heated air-circulation unit (Werner Mathis AG, Oberhasli, Switzerland).

Samples were designated according to the amount of SWCNTs added and the printing dispersion/paste, where 0.1, 0.2 or 0.4 denotes the addition of SWCNTs (0.1 wt%, 0.2 wt% and 0.4 wt%, respectively) and the slash (/) separates the first imprint from the second (for example, PCNF + 0.4 SWCNT/AP means printing of 0.4 wt% SWCNT dispersed in PCNF as the first print/layer, followed by AP printing as the second). Referenced samples were noted as Ref (non-printed), AP (solely AP printed), AP + Disp (solely SWCNTs-dispersing agent within AP), PCNF + Disp/AP (printing of solely SWCNTs-dispersing agent within PCNF, followed by printing of AP). The AP pastes were always printed on the fabric back-side (designated as B), while the PCNF dispersions were always printed before the AP (as the first print or layer) and on the fabric front-side in a two-layer process (designated as two-layers/F + B), or on the back-side of the fabric in a two-layer process (designated as two-layers/B).

### 2.4. Fabrics’ Washing and Drying

After the treatment, all the samples were washed once in the washing machine at 60 °C and centrifuged at 1000× *g* rpm (Gorenje SensorCare, model W8665K, Velenje, Slovenia; ISO 6330:2012 [31]) using detergent without bleaching agents (IEC BASE A; 66 g/2 kg) and then dried at a low temperature in a domestic drying machine (Gorenje SensorCare model D82426, Velenje, Slovenia). 

### 2.5. Fabric’s Analysis

The samples were conditioned for 24 h at 20 ± 2 °C and relative humidity of 65 ± 5% (ISO 139:2005 [32]) before the testing, unless other methods’ standard conditions were used during the analysis. 

Scanning Electron Microscope (SEM) imaging was performed by a low vacuum microscope (FEI Quanta 200 3D, Thermo Fisher Scientific Inc., Waltham, MA, USA) to evaluate the print’s structure, homogeneity/distribution and durability. 

The colour values of differently treated samples were determined by reflectance measurements in the wavelength range of 400–700 nm, using a two-rays spectrophotometer, Spectraflash SF 600 PLUS (Datacolor Inc., Lawrenceville, NJ, USA), equipped with an Ulbricht sphere and measuring geometry of d/8 ° under a standard illuminant D65 (LAV/Spec. Incl.). The colour strength (K/S) values were calculated from the reflectance curves according to the following equation: $K/S=\frac{{\left(1-R\right)}^{2}}{R}$, where K is the light absorption coefficient, S is the light scattering coefficient, R is the decimal fraction of the sample’s reflectance. The CIE total colour differences (dE*) of differently-printed and reference samples were obtained from all three coordinate differences (dL*—the difference in the brightness, da*—the difference at the red/green axis, and db*—the difference at the yellow/blue axis) using the following equation: dE* $=\sqrt{{\left({\mathrm{dL}}^{*}\right)}^{2}+{\left({\mathrm{da}}^{*}\right)}^{2}+{\left({\mathrm{db}}^{*}\right)}^{2}}$. 

The fabric’s mass change (Δm) was assessed using the following equation: Δm = *m*_i_ − *m*_c_, where *m*_i_ and *m*_c_ are the masses of samples before and after the treatment, respectively. The average value of at least three measurements was conducted for each sample. 

The thickness (ISO 5084:1996 [29] using a Thickness gauge for Textiles and Rubber, Hildebrand GmbH, Wendlingen am Neckar, Germany), dimensional change (ISO 5077:2007 [33]), air permeability at pressure difference of 100 Pa (ISO 9237:1995 [34]) using a Karl Schroeder KG Air Permeability Tester D-6940, Weinheim, Germany), were assessed according to the relevant Standards. 

The hydrophilicity of the samples and their water absorbency (ASTM D5725-99 [35]) was determined by measuring the Contact Angle (CA) of a 3 mL milliQ water droplet and the time of its stay on the surface using the goniometer (OCA 35 model, Dataphysics, Filderstadt, Germany). Between four and ten measurements were performed at room temperature and on both sides of the samples, and being given as the mean values. In addition, the water absorption capacity (WAC) was evaluated by immersing the 30 × 30 mm sample in mQ water for 60 s, then placing it on a filter paper for 10 s for drainage, and weighed; the WAC was calculated according to the following equation: $\mathrm{WAC}=\frac{B-A}{A}\cdot 100$, where A is the specimen weight before immersion and B is the specimen weight after immersion in water. At least four measurements were taken for each sample.

The thermophysiological comfort properties were evaluated by measuring the thermal resistance and water-vapour resistance, and recording a warm-cool feeling, according to ISO 11092:2014 [36] using the measuring instrument KES-F7 Thermo Labo II (Kato Tech Co- Ltd., Kyoto, Japan), where the heat flow was measured through the sample. Using the dry contact method, the samples’ thermal resistances were obtained by exposing them to a dry heat flow in response to a steadily applied temperature gradient. The same procedure was used with a wet filter paper on the heated plate for evaluating the water-vapour resistance, where the evaporative heat flow was determined by the steadily applied water-vapour pressure gradient. Both were calculated from the measured heat flow, by the following equations: $R_{\mathrm{ct}}=\frac{\left(T_{s}-T_{a}\right)\cdot A}{\phi_{\mathrm{ct}}}$ and $R_{\mathrm{et}}=\frac{\left(p_{s}-p_{a}\right)\cdot A}{\phi_{\mathrm{et}}}\;$, where R_ct_ is the thermal resistance of the textile material (m^2^K/W), R_et_ is the water-vapour resistance of the textile material (Pa m^2^/W), A is the area of the heat plate (m^2^), T_s_ and T_a_ are the temperatures of the heat plate (35 ± 1 °C, skin temperature of a human body) (K) and air temperature (K), p_s_ and p_a_ are the saturated vapour pressure on the heat plate (Pa) and vapour pressure in the tunnel (Pa), ϕ_ct_ and ϕ_et_ are the corresponding heat flows (W). The warm-cool feeling (q_max_) was recorded as the maximum level of heat flow required per unit area (W/cm^2^). Five measurements were performed to obtain q_max_, six measurements for R_ct_ and four measurements for the R_et_ values.

Thermo-Gravimetric Analysis (TGA) and Differential Scanning Calorimetry (DSC) tests of the samples were performed under an air atmosphere in a temperature range from 25 to 600 °C and a heating rate of 10 °C/min, using a 50 mL/min of flow rate and a DSC1 analyser (Mettler Toledo, Greifensee, Switzerland).

The flame resistance of the treated samples was evaluated by the ease of ignition according to ISO 15025. The back-side of the vertically aligned samples was exposed to an open flame for 10 s, directed perpendicularly towards the fabric, in both directions. In the case of ignition, the burning time was recorded. Three measurements were taken for each sample.

The Ultraviolet Protection Factor (UPF) was assessed according to Standard (EN 13758-1 [37]) using spectrophotometer UV/VIS/NIR (Perkin Elmer, Waltham, MA, USA), by recording the transmission values in the range of 290–400 nm at every 5 nm. For each sample, three measurements of transmittance were recorded, then the UPFs of individual samples were calculated using the following equation: $\mathrm{UPF}=\frac{\sum_{\lambda =290}^{\lambda =400}E\left(\lambda \right)\varepsilon \left(\lambda \right)\Delta \lambda }{\sum_{\lambda =290}^{\lambda =400}E\left(\lambda \right)\varepsilon \left(\lambda \right)\Delta \lambda }$, where E(λ) is solar irradiance, ε(λ) is the erythema action spectrum, Δλ is the wavelength interval of the measurements, and T(λ) is the spectral transmittance at wavelength λ. The ranges of protection are categorised from good (UPF 15–24), very good (UPF 25–39) to excellent (UPF 40–50, 50+), where fabrics with UPF less than 15 are not labelled.

The electrical resistance (R) of the samples was measured using an impedance analyser (Agilent 34410A 6 ½ Digit High Performance Digital Multimeter, Agilent Technologies Inc., Santa Clara, California, USA), using two parallel copper electrodes being in contact with the sample, at three measuring points. The electrical resistivity (ρ) was calculated according to the following equation: $\rho =R\frac{t\cdot w}{l}$ [Ω m], where l is the length of the sample (m), t is the thickness (m), w is the width (m), and R is the resistance (Ω). The electrical resistivity was used to calculate the electrical conductivity (σ) according to the following equation: $\sigma =\frac{1}{\rho }$ (S/m). 

Tensile strength and elongation (ISO 13934-1 [38]), together with tear strength ISO 13937-2 [39]), were evaluated using a Tinius Olsen testing machine, H10KT (Tinius Olsen Ltd., Redhill, UK), with a 1 kN load cell. Testing was conducted in both weft and warp directions, where all the samples were preconditioned prior to analysis. For tensile testing, the samples were cut into dimensions of 250 ± 2 mm × 50 ± 1 mm. On the other hand, tear strength analysis was performed at a gauge length of 200 mm, at a constant rate of extension of 100 mm/min, and preloading of 2N. Samples undergoing the tear strength analysis were cut to the size of 200 ± 2 mm × 50 ± 1 mm. Afterwards, from the centre of the width, a longitudinal cut was made with a length of 100 ± 1 mm. The final position of the test completion was marked on a length of 25 ± 1 mm from the middle of the uncut sample side. For both analyses and both directions, five samples were analysed and average values were expressed.

The abrasion resistance of the samples was determined using a Martindale M235 Abrasion and Pilling Tester (SDL Atlas Textile Testing Solution, Shenzhen, China) according to ISO 12947-2 [40]. Samples were cut into circles with a diameter of 38 mm. Twelve kPa pressure was applied to each sample, and the front-sides of the fabrics were exposed to an abrasion test. All the samples were weighed before analysis, after a certain number of abrasion cycles and at the end of testing, which determined the weight loss until the breakage (ISO 12947-3 [41]). Breakage was acknowledged when at least two threads were torn. Visual changes that occurred because of the abrasion test (lustre, colour, surface nap or pile, pilling, matting) were assessed according to ISO 12947-4 [42]. Four tests were conducted for each sample.

## 3. Results

### 3.1. The Coating’s Patterning and Imprinting

**Figure 1:**
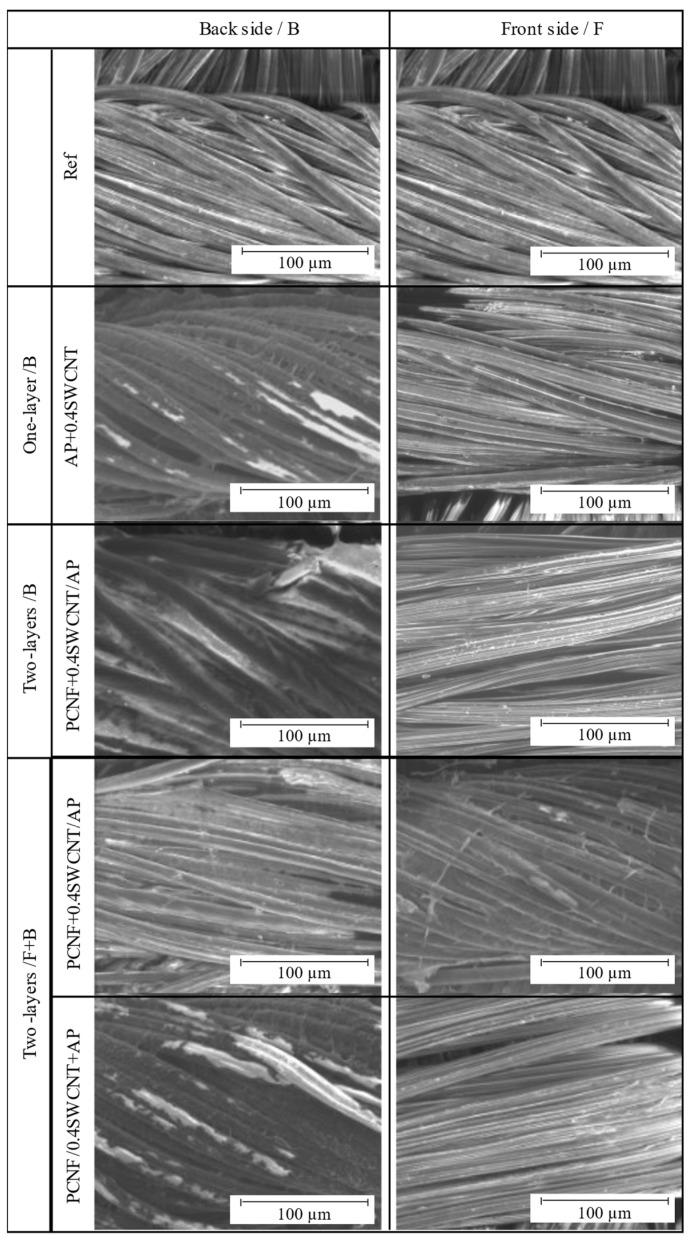
SEM images of the back/B and front/F sides of non-treated (Ref) and selected differently printed fabrics.

The patterning and structuring of SWCNTs-containing PCNF dispersions and AP pastes on the fabric surfaces (both front- and back-sides) using different printing strategies (printed on the back-side as one or two layers, or on both sides as one-layer), and their imprinting, were assessed by SEM imaging (Figure 1) after standardised laundry washing. Although some coatings were slightly washed off, it was still possible to observe a homogeneous and evenly distributed pattern deposition over the entire fabric’s surface, regardless of the type of coating (PCNF or PA, with and without the addition of SWCNT) and printing strategy. Yarns appeared smooth and regular, but slightly glued after AP deposition, and some rare spots of AP were observed on the unprinted/front-side (not presented). On the other hand, the addition of SWCNTs to AP (sample AP+0.4SWCNT) was reflected in a darker colour of the deposited patterns, which, however, could not be observed on the front-side, indicating that such a coating was mostly adhered and cross-linked only on the printed side. In the case of two-layer back-side printing, where the PCNF was printed as the first layer under the AP, the empty spaces between threads were almost completely filled (not presented), which was even more pronounced with the addition of SWCNTs’ (PCNF + 0.4SWCNT/AP); and again, there was almost no coating seen on the unprinted/front-side. On the other hand, rare white spots and glued yarns could be observed on the fabric’s back-side in the case of applying solely PCNF under the AP in two-sided printing (not presented). The presence of PCNF (containing SWCNTs or not) was also observed, even if applied on the other (front) side than the AP, meaning that it was attached to the fabric permanently by AP’s penetration through the fabric and co-crosslinking it. However, the addition of SWCNTs into the AP in case of two-sided printing (PCNF/0.4SWCNT + AP) resulted in a much darker and homogeneous colour of the deposited patterns as compared to their counterpart (adding SWCNTs to PCNF; PCNF+0.4SWCNT/AP), confirming their insufficient fixation within PCNF and removal during washing.

**Figure 2:**
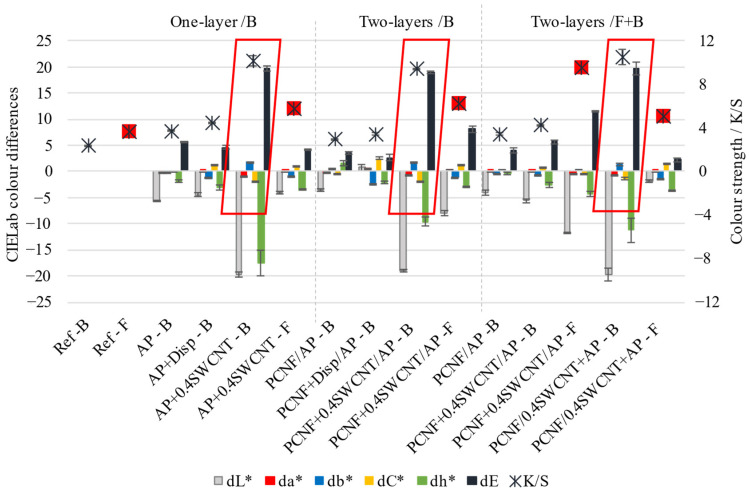
The effect of printing on the fabric’s colour strength (K/S) and CIEL*a*b* colour differences in lightness (dL*), saturation (dC*), red–green (da*) and yellow–blue (db*) axes, as well as total values (dE), determined on the back/B (turned outwards) and front/F-side (facing the wearer) of the fabric.

The colorimetry was used in addition to evaluate the SWCNTs’ coating efficacy and its penetration on the non-printed side, by determining the colour strength (K/S) and CIELab colour differences, presented in Figure 2. As seen from the graphs, the K/S values of SWCNTs-printed sides of the fabric were increased from about 2.4–3.7 (Ref) to about 10.1–10.5, and reduced back to about 6.2–5.1 on the other side, regardless of the method of SWCNTs’ application (within PCNF or AP); a similar trend was observed for the lightness (dL), hue (db) and total colour difference (dE) values. It can also be observed that the SWCNTs-dispersing agent (AP + Disp) did not contribute to the colour changes, but only slightly the AP and PCNF, which can be related to the less effective coating. The highest K/S (from 10.5 on the back-side to 5.1 on the front-side) and total colour difference (from dE = 19.8 on the back-side to dE = 2.23 on the front-side), was obtained when SWCNTs were applied through PCNF in a two-layer process, printed on the fabric’s back-side (PCNF+0.4SWCNT/AP). Slightly higher K/S and dE values (thus, more SWCNTs present) were observed for the non-printed side when applied within PCNF, where, on the contrary, their applying within AP did not show any difference as compared to the reference sample. It can be confirmed that SWCNTs can be applied with AP and PCNF, wherein such a coating prefers to remain on the surface of the fabric after AP crosslinking, rather than penetrating through the fabric to the other side, probably due to the different surface tension properties of SWCNTs containing dispersion/paste, and their adhesion to the fabric as if they did not contain them. The results coincide well with the SEM analysis presented on Figure 1.

### 3.2. Thickness, Mass Change, Dimensional Change and Air Permeability

The thickness, coating’s mass change values, dimensional change and air permeability of the fabrics have been assessed to determine the effect of both SWCNTs and PCNF, as well as the printing strategy on the printing efficacy. 

**Figure 3:**
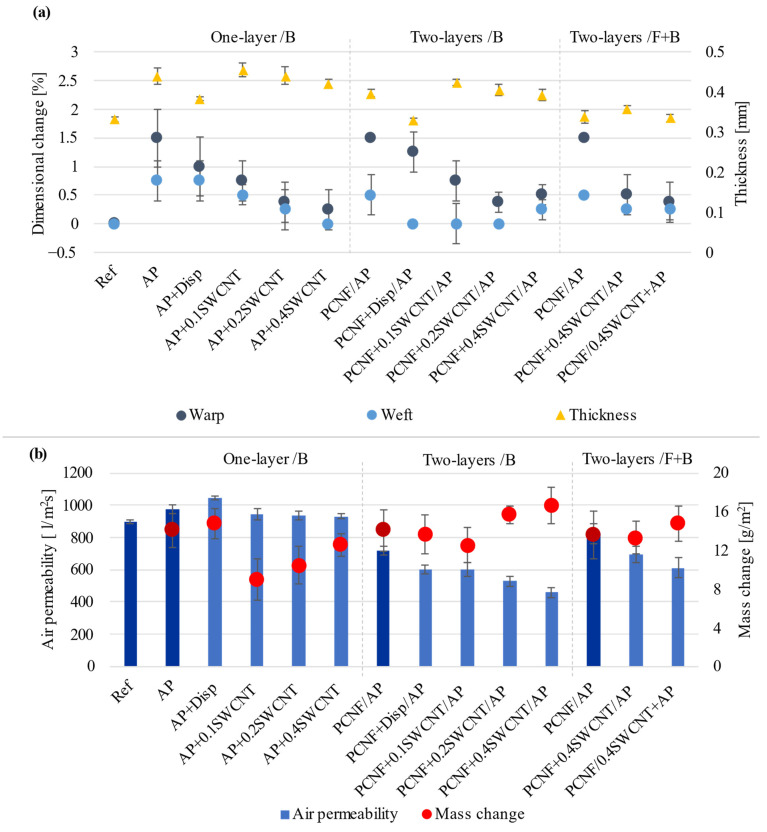
The effect of printing on the fabric’s: (**a**) Dimensional change and Thickness, and (**b**) Air permeability and coating’s Mass change values.

As presented in Figure 3a, the printing of AP increased the thickness of the fabric by around 32% (from 332 μm to 439 μm), being related to the deposition of AP reflected in a mass change (14 g/m^2^), and an increase of the air permeability (Figure 3b) (by 8.3% to 973 L/m^2^s) as compared to the non-treated (Ref) sample (898 L/m^2^s), which was additionally increased using SWCNTs-dispersing agent (to about 1044 L/m^2^s). Such properties may be due to the fabric’s dimensional change (Figure 3a). While the non-treated sample did not shrink after being washed and dried, all other samples shrank between 0.25–1.5% in the warp direction, and it was generally below 0.75% in the weft direction, due to different prints’ deposition and imprinting. Another important parameter is the fabric’s woven structure, where weft-directed threads (made of hydrophobic meta-aramid/acrylic and FR viscose fibre blends) were highly subjected to the coating when compared to warp-directed threads (made with the presence of hydrophilic viscose fibres on the front-side). Such a construction not only dictates the efficiency of printing and adhesion to the fibres, but, above all, the AP mass change and its imprinting, which influence the threads’ reinforcement, due to their crosslinking with AP and consequently dimensional change of the fabric, being more obvious in the more open/less dense (36 vs. 51 threads/cm) warp-directed threads. The highest dimensional change (about 1.5%) was, thus, obtained in the warp direction for the samples printed with pure AP (AP, PCNF/AP, PCNF + Disp/AP).

The air permeability of samples printed with AP containing SWCNTs was around 930 L/m^2^s and almost independent of the SWCNTs’ content, while the thickness was reducing slightly (from about 0.45 mm to 0.42 mm) and the mass change increasing (from about 8.9 g/m^2^ to 12.6 g/m^2^) with an increase of SWCNT, meaning worse crosslinking of AP in the presence of the SWCNTs and its lower deposition. The pre-printing of PCNF in two-layer one-sided printing reduced the air permeability significantly (to 717 L/m^2^s), being even more pronounced with added 0.4 wt% SWCNTs to PCNF (up to 459 L/m^2^s or 49% reduced). Such a reduction was related to the high adhesion of PCNF, and, consequently, the highest mass change among the samples (an increase to 16.6 g/m^2^) without affecting the dimensional stability significantly (0.5% warp, 0.25% weft). The hydrophobic nature of the SWCNTs–PCNF mixture affected the crosslinking and adhesion of AP on such an interface, which, thus, increased its deposition (as also presented on the SEM images, Figure 1). 

In the case of two-sided PCNF/AP printing, the air permeability of the fabric was higher (824 L/m^2^s) than for the one-sided two-layered (717 L/m^2^s) printed sample at similar mass (13.6 g/m^2^ and 14.0 g/m^2^) and the same dimensional change (1.5% warp and 0.5% weft), but slightly lower thickness (0.33 mm and 0.39 mm). The presence of SWCNTs affected the air permeability more when printed within AP (611 L/m^2^s) than PCNF (695 L/m^2^s), while giving a higher mass change (14.7 g/m^2^) with insignificant dimensional change, again indicating a better adhesion of AP with a hydrophobic meta-aramid and FR viscose fibres. 

### 3.3. Water-Vapour Resistance, Surface Wetting and Water Adsorption Capacity

When a fabric is worn next to the skin, it must also absorb sweat/moisture and allow its evaporation away from the body and through the fabric [43], thus keeping the wearer’s body in thermal equilibrium [44,45]. Otherwise, the accumulation of moisture next to the body not only creates a sticky feeling and discomfort [46], but is also a breeding ground for the growth of many different types of microbes [47]. The water-vapour resistance, surface wettability and water absorption capacity of the fabric were thus performed to access the impact of different printing processes on the moisture management regime.

**Figure 4:**
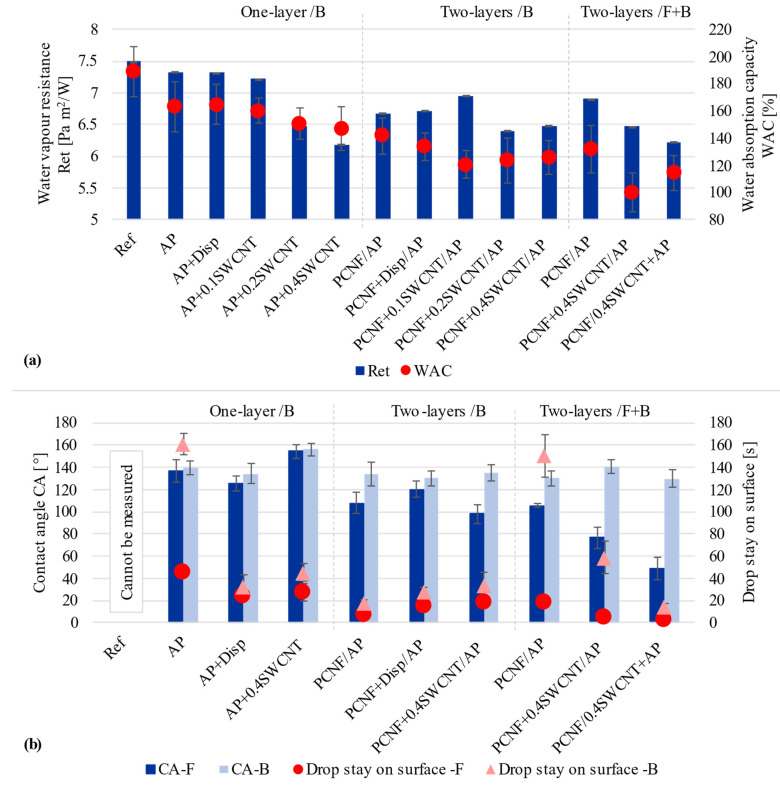
The effect of printing on the fabric’s: (**a**) Water vapour resistance determined on the front/F side and Water absorption capacities, and (**b**) Values of the Contact Angle (CA) and duration of the water droplet’s stay on the sample’s surface, measured on the fabric’s front/F and back/B sides.

As seen from Figure 4a, the non-treated and AP treated fabrics had water vapour resistance values of about 7.49 Pa m^2^/W and 7.33 Pa m^2^/W, with water absorbency of 188% and 163%, respectively. The addition of SWCNTs-dispersing agent in the AP had no impact on both values, while the addition of SWCNTs further reduced the fabric’s water vapour resistance by up to 18% (to about 6.18 Pa m^2^/W), being accompanied by about 41% lower water absorption capacity. By applying solely PCNF under the AP, the resistance values were again increased slightly to about 6.67 Pa m^2^/W, or 6.9 Pa m^2^/W in the case of both-sided printing, being accompanied with further lowered water absorption by 47% for one-sided and 62% for both-sided printing. Both-sided printing of fabric gave even lower values, where about 6.22 Pa m^2^/W and 100% water absorption capacity were obtained for the sample where SWCNTs were applied within AP. Such a decrease in water vapour resistance could be regulated by both the change of surface roughness, where the assembly of SWCNTs formed nanoscale surface roughness when applied on the fabric’s fibres, and lowered the surface energy [17], which decreased the apparent wetting angle and allowed faster water spreading [48], as well as due to the presence of PCNF, which provides different diffusion along the fibres and their wetting (as already found in our previous study using microfibrillated cellulose [25]) in addition to hydrophilic viscose fibres, being more pronounced on the fabric’s front side.

The wettability of the fabrics was evaluated on both surface sides by measuring the CA of a milliQ water drop in the moment of the contact and the time of the droplet’s stay on the surface (Figure 4b). As seen from the graphs, the non-treated fabric was completely hydrophilic, since water was absorbed instantly on both sides, and the CA was impossible to measure. The printing of AP turned both fabric surfaces into entirely hydrophobic (CA ≥ 136°), confirming that the APs diffuse and penetrate through the fabric, and also covered the non-printed side, although the water drop penetrated faster on the front/un-printed side (46 s) as compared to the back/printed side (161 s). The addition of SWCNTs-dispersing agent to AP reduced the CA of both sides of the fabric slightly (125°/front, 134°/back), while the simultaneous presence of SWCNTs increased it to above 154° on both sides, and the time of drop penetration was reduced for both samples to about 25 s/front- and 35 s/back. Such a high CA makes the fabric superhydrophobic (CA > 150°), owing to the CNT aligned nanostructures with hydrophobic surface properties [17,23]. The CA values on the front-side of the fabric were reduced (107°) in the case of two-layer one-sided printing using PCNF as the first layer, resulting in a much faster (9–17 s) drop’s penetration, being reduced further to about 98° when SWCNTs were added into the PCNF, and being accompanied by a slight reduction of CA also on the fabric’s back/AP printed side (133°). For two-sided printing, the hydrophobicity (CA of 130–140°) of the fabric’s back-side was not affected by the addition of SWCNTs, but it was highly decreased on the front-side (to 76°) when SWCNTs were applied though the PCNF, and, further, to 51° when applied within AP, which turned this side hydrophilic. Such treated fabrics thus resulted in hydrophilic front-sides, where excessive water could be absorbed quickly and transferred further away from the skin through the fabric (as already discussed by water-vapour resistance analysis, Figure 4a), and, at the same time, improving the hydrophobic outer side with the effect of water repellence. 

Our research gained comparable results with other studies, since the CA of one-layer printed fabrics was >154° (on both sides), meaning that the limit was reached for super-hydrophobicity. The CA of the back-sides of two-layer printed samples were just below the limit of super-hydrophobicity, but still highly hydrophobic (130–140°). Other authors achieved CAs above 150° when, for example, dip-coating cotton fabrics with MWCNTs and aromatic azide polymer [17], or MWCNTs and butyl acrylate [15,16], or using poly(ethylenimine), ammonium polyphosphate and CNT in a layer-by-layer assembly process [21]. Superhydrophobic coatings have hardly been used, because the moisture management properties of the fabric can be deteriorated significantly after such a modification. In this regard, there have been rare studies [45,49] that developed textile coatings which resulted in a material with asymmetric wettability, i.e., one side of the fabric surface to be hydrophobic and the opposite surface to be hydrophilic. Kwon [45] fabricated a superhydrophobic lyocell fabric in which CA exhibited greater than 161°, where the other side was super hydrophilic, but able to absorb even or about 117% of moisture. Those results came close to ours (PCNF + 0.4SWCNT/AP), where the fabric’s back-side CA was around 141° and the front-side 76° while absorbing around 100 % of moisture.

### 3.4. Thermal Resistance and Corresponding Cold-Warm Feeling Properties

**Figure 5:**
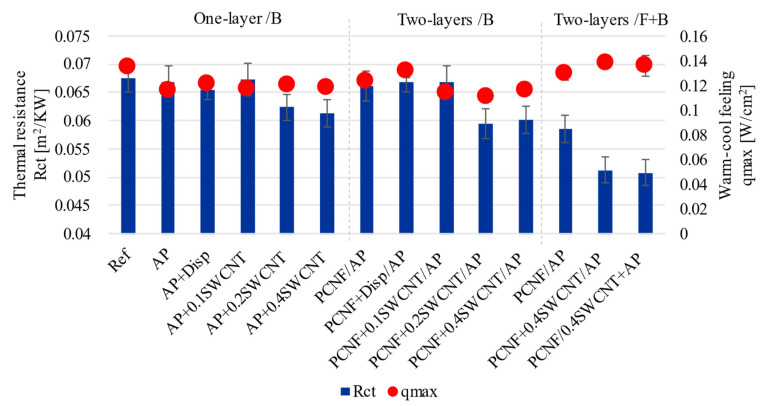
The effect of printing on the fabric’s thermal resistance and corresponding cold-warm feeling, conducted from the front/F side (being in contact with the skin) at 35 ± 1 °C.

The thermal resistance (R_ct_) properties and instant warm-cool feeling (q_max_) were evaluated for determining whether SWCNT treated fabrics possessed the effect of cool feeling. As seen from Figure 5, the q_max_ values decreased with the printing of AP by about 17% and pre-printing with PCNF by 10% towards a warmer feeling (from 0.134 W/cm^2^ to 0.115 W/cm^2^ and 0.122 W/cm^2^, respectively). Values were not affected further with the addition of SWCNTs-dispersing agent (0.121 W/cm^2^), nor with the addition of SWCNTs to AP (0.118 W/cm^2^), or to PCNF (0.116 W/cm^2^), when applied as one-sided printing. In the case of two-sided printed fabrics, the q_max_ rose up to 0.138 W/cm^2^, which was close to the value of 0.14 W/cm^2^ that denotes the cold feeling sensation. However, much higher differences were present when measuring thermal resistance, which got decreased up to 11% (from 0.067 m^2^K/W to 0.060 m^2^K/W) for one-sided SWCNT printing, and, further, up to 0.051 m^2^K/W when printed both-sided, independent of whether SWCNTs were dispersed in AP or PCNF, meaning about an 25% reduction of thermal resistance. Such a reduction was due to the PCNF and SWCNTs depositions, which covered the empty spaces on the fabric’s surface, together with a reduction of the air gaps inside the fabric, resulting in higher thermal conductivity [50]. Decreased thermal resistance also owed to the thermally anisotropic CNTs, as each tube can transfer heat longitudinally while being relatively insulative in its diameter. This means that patternly deposited CNTs are able to conduct heat through the fabric, which protects the wearers from exhaustion and heat stress [23], since excessive heat is transferred away from the body to the outer side. An approximately 25% improvement of thermal conductivity by using only 0.4 wt% of SWCNTs was a significant achievement compared to other studies. Abbas et al. [10] improved the thermal conductivity of cotton up to 132% (from 0.047 to 0.072 W/mK) using 11 w% SWCNT content, while keeping its hydrophilicity and air permeability. An about 60% increase (from 0.027 to 0.045 W/mK) of thermal transfer was obtained by Rahman et al. [14] using 1 wt% of citric-acid functionalised MWCNTs.

### 3.5. Thermal Stability and Flammability

**Figure 6:**
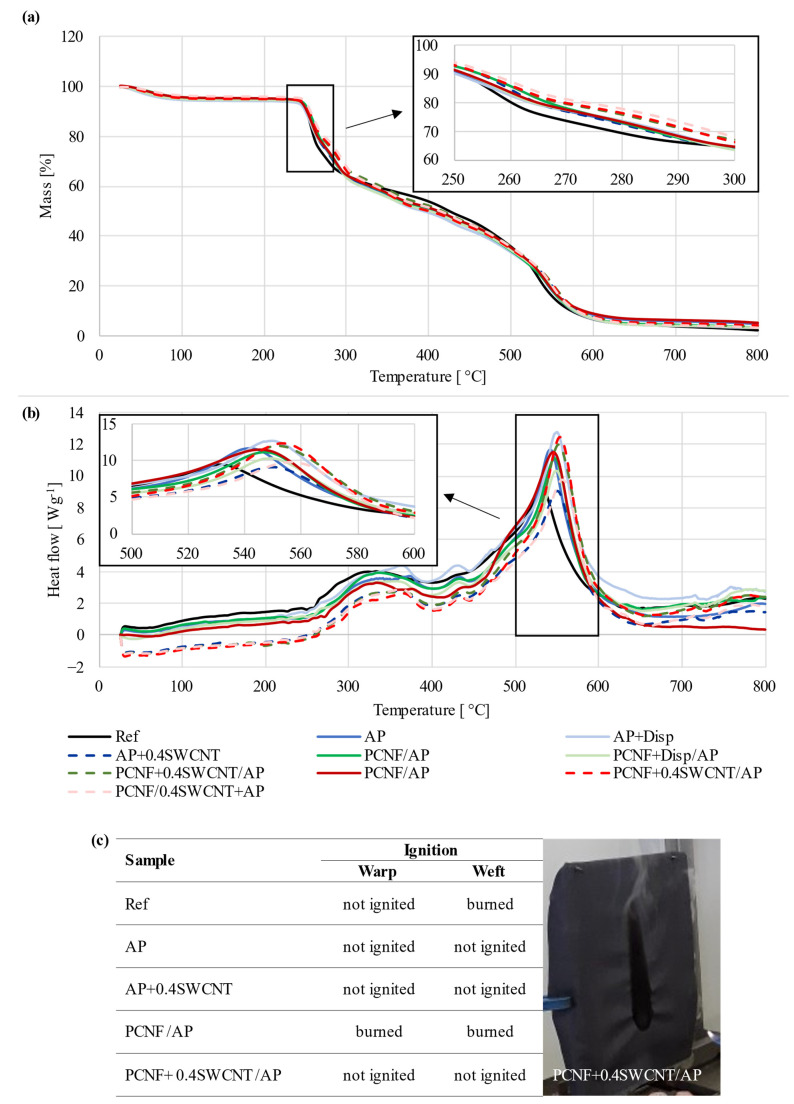
(**a**) The effect of printing on the fabric’s: (**a**) Thermogravimetric (TGA), (**b**) Differential scanning calorimetry (DSC), and (**c**) Flame-resistant analysis.

TGA and DSC measurements were conducted to identify the effect of differently printed fabric on thermal degradation behaviour and corresponding enthalpy changes. As can be seen from Figure 6a, three mass loss stages are present for the degradation of all samples. The first mass loss (of about 5%) appeared between 40 °C and 120 °C, which represents the evaporation of the absorbed water. The 38% mass loss in the range of 230 °C–310 °C was rapid, and corresponded to the degradation of the viscose [51], being also observed as the first broad exothermic peak on the DSC thermogram (Figure 6b). The following step started at around 375 °C and belonged to the melting temperature of the meta-aramid, with the degradation temperature around 500 °C [52], where around 20% mass was lost. From 500 °C, the mass loss was rapid, corresponding to the final decomposition of the viscose, FR viscose and meta-aramid, given the sharp exothermic peak, representing about 95% mass loss up to 600 °C, leaving almost no residue at the final 800 °C. When the viscose started to degrade (in the range of 260–300 °C), the degradation temperatures were shifted slightly towards higher temperatures for all coated samples as compared to the reference, with 30% mass loss around 277 °C. For the AP (with/without SWCNT-dispersant treated samples) printed sample, such an amount of mass loss was moved to around 283 °C, for PCNF pre-printed to around 285 °C, while for the samples containing SWCNTs, these temperatures were moved to around 284–292 °C and, further, to 292–295 °C, by being applied through AP or PCNF in one side and both side printing processes, respectively, meaning up to around 18 °C of temperature shift. Even higher temperature shifting was observed for the second exothermic peaks in the range of 500–600 °C, being related to the degradation of meta-aramid fibres, which was shifted from 530 °C up to 557 °C for both side printed samples (PCNF/0.4SWCNT+AP), where the PCNF was applied on the front-side and SWCNTs through the AP on the fabric’s back-side, meaning even a 26 °C temperature shift towards higher values. Such protection was due to the CNTs’ flame retardation mechanisms, where the formed char creates a heat barrier that delays degradation by radiating heat back into the gas phase [2]. The additional effect could be due to the presence of phosphor groups on CNF, which might have led to the formation of stronger and more cohesive char, since phosphor has the ability to trigger and promote the char formation [53]. 

The flammability test was also performed (Figure 6c) by monitoring the ease of ignition when exposing samples to the open flame in both the weft and warp directions for 10 s. The non-treated sample burned in the weft direction, probably due to the presence of viscous filaments. When applying PCNF, the sample also burned in the warp direction, while all other samples treated with SWCNTs did not ignite after being in contact with the open flame. 

### 3.6. UV Protection Properties

UV radiation can cause skin damage (cancer, burns) [22], induce photoallergic reactions and degradation of polymers, due to photochemical reactions within their structure [16]. CNTs have the ability of high UV-blocking improvements of materials, including textiles, which is caused by the small diameter of carbon atoms and strong C-C bonding together with easily accessible free electrons of p-bonds [22]. Liu et al. [16] showed that CNT treatment of cotton fabric endows fabrics with good UV-blocking properties, where the UPF factor increased from 60 to 246 with an increase of CNT content from 0.25% up to 2.5% [16]. Mahmoudifard et al. [22] obtained a UPF of 78 by treating cotton fabric with SWCNTs, showing that SWCNTs gave higher protection than MWCNTs and also higher than TiO2 and ZnO as the most common used inorganic UV absorbents. Aniline functionalised MWCNTs [11] induced the fabrics with a UPF up to 173, being applied as 1 wt% concentration. 

**Figure 7:**
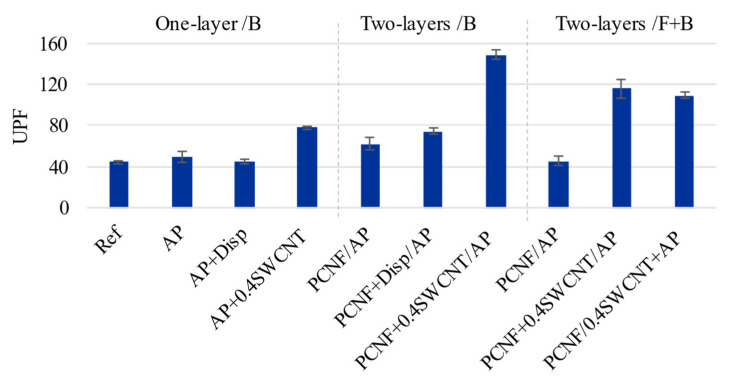
The effect of printing on the fabric’s ultraviolet protection factor (UPF) properties.

Our results showed a similar trend (Figure 7). The non-treated fabric had a UPF of 44, which was increased to 62 by applying PCNF, while the contribution of the AP or SWCNT-dispersing agent was insignificant. The UPF was further increased to around 148 when SWCNTs were added to the PCNF in the case of one-sided two-layers’ (PCNF ± SWCNT/AP) printing. On the other hand, when SWCNTs were applied through the AP in a both-sided printing, the UPF was reduced to about 109 (PCNF/SWCNT + AP) and 116 (PCNF + SWCNT/AP), which might have been due to the worse crosslinking of the AP in the presence of SWCNTs, and in the case of two-sided printing, with consequently lower amounts applied as already established by other studies (higher air permeability, as well as coating’s thickness and mass change, Figure 3) being lower than those of one-sided printed samples.

### 3.7. Electrical Conductivity 

**Figure 8:**
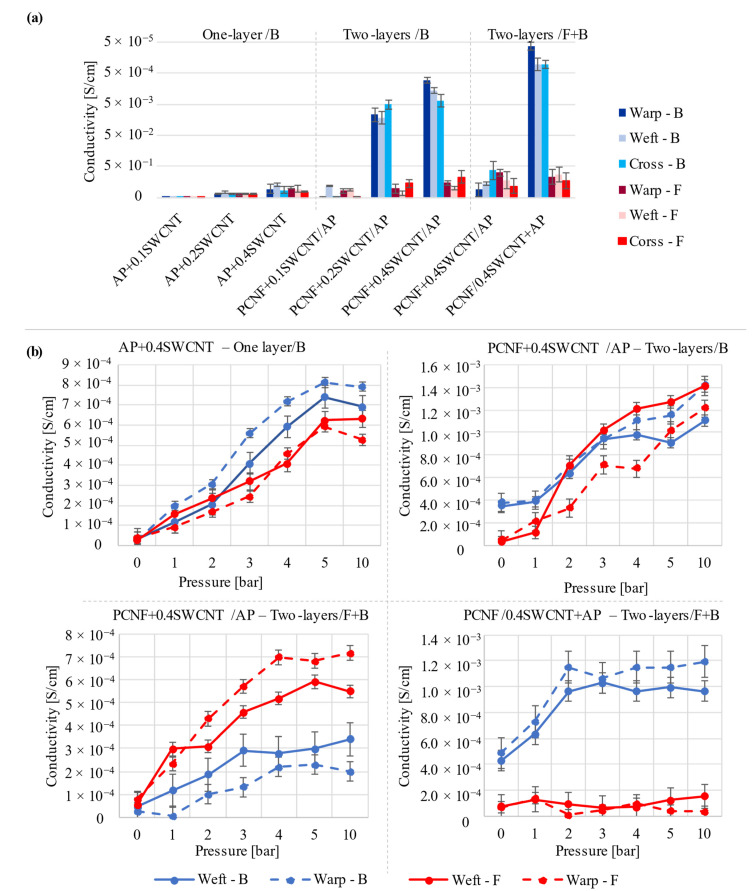
Electrical conductivity of SWCNT treated fabrics, evaluated on both back/B and front/F sides, (**a**) Without and (**b**) With applied different pressures.

The electrical resistance of the material shows the ability to resist the electrical current through or along it. The electrical conductivities of fabrics are, thus, affected by the uniformity of the CNTs deposition and its construction [54]. Since the SWCNTs were applied on the fabric by different printing strategies (front and back) and the construction of the fabric was different on different sides, the electrical resistivity was measured on the samples front- and back-sides, as well as in the warp (20 cm) and weft (20 cm) directions and diagonal-cross (28.3 cm), and corresponding conductivities calculated related to this. The samples treated without SWCNTs were not electrically conductive, also the one printed with the SWCNT-dispersing agent, therefore, they are not presented in the graphs (Figure 8a). On the other hand, the conductivities of SWCNTs printed samples were generally increasing with an increase of SWCNTs applied, being in the range from 2∙10^−8^ S/cm up to 4.9∙10^−4^ S/cm. One-layer back-side printing (AP + SWCNTs) gave generally much lower conductivities, up to 3.9∙10^−5^ S/cm, which could be the cause of lower SWCNT deposition (due to the worse crosslinking of the AP in the presence of SWCNTs and its deposition, Figure 3b) and possible imprinting into the fabric microstructure by showing similar values on both sides of the fabric. On the other hand, the conductivity was increased significantly (up to 3.5∙10^−4^ S/cm) on the fabric’s back-side when SWCNTs were applied through PCNF pre-printed in a two-layer process (PCNF+SWCNT/AP), given the much lower values on the fabric’s front-side (up to 2.8^−5^ S/cm). This effect was, surprisingly, increased further in the case of both-sided printing, when PCNF was applied on the front-side and SWCNTs through the AP on the back-side (PCNF/ SWCNT+AP), reaching conductivity of 4.9∙10^−4^ S/cm. It can be concluded again that PCNF blocked the SWCNT+AP coating from being pushed onto the other side of the fabric, thus resulting in more surface printing, which improved the AP crosslinking, resulting in a higher mass change and lower air permeability (Figure 3b), and, consequently, making the back-side the most electrically conductive among the samples, while not affecting the front-side’s conductivities (2.4–7.9∙10^−5^ S/cm). 

There were also slight differences in the conductivity values, depending on the measuring line (weft and warp directions), which might be related to the inhomogeneous and anisotropic fabric surface structure on one side, and inhomogeneous coating of the deposited SWCNT patterns across the fabric’s surfaces, being measured on the other. Slightly higher conductivities were observed for measurements in the warp direction, which might be related to the slightly higher dimensional change of the sample in that direction (Figure 3a) due to the denser fabric structure, and better adhesion of the coating on the fabric’s back-side, where mixed yarns of meta aramid and FR Lenzing viscose predominated.

Although the obtained conductivities were quite low, they were comparable to the results of other studies using CNTs in combination with different chemistry (dispersants, fabric pre-treatments) and other coating techniques (immersion, dipping–drying, coating at different temperatures, spinning), being studied to improve their dispersibility and homogeneous crosslinking with the textile. For example, Nafeie et al. obtained electrical conductivities up to 2∙10^−3^ S/cm (for non-washed samples) using 5 g/L of carboxylated MWCNTs in the presence of citric acid and sodium hypophosphite on an oxidation pre-treated wool fabric [18]. Similar conductivities (3.5∙10^−3^ S/cm and 2.5∙10^−3^ S/cm) were recorded by Krucinska et al. [5] and Rahman et al. [14] by using 1.5 wt% of MWCNT, together with the aliphatic urethane acrylate and citric-acid-assisted plasma functionalisation processes, respectively. On the other hand, much higher conductivities were obtained by using polyester yarns coated with polypropylene/MWCNTs in a melt extrusion process (0.8 S/cm, [19]), and regenerated cellulose fibres reinforced with MWCNT by wet spinning (2.7 S/cm, [24]). 

The literature shows that the electrical properties of textile materials are sensitive to applied pressure, since the change in electrical resistance occurs between two surfaces in accordance with the applied load, due to deformation of the geometry of the material [55], although comparable studies are hard to find, since different electrical properties are involved, like resistance, capacitance, impedance, charge displacements [56]. Euler et al. [57] found that the resistance of polyester with integrated silver-plated polyamide yarns got reduced by up to 45% with applied pressure of 1000 g, where the relative change was bigger for a bigger pressure change, but not linear. Giovanelli and Farella [56] printed conductive ink electrodes on a PET substrate, and tested its response to pressure in the range 0.9–2.7 kPa, with changes in conductance from 0.7 mS to 1.6 mS. Tseghai et al. [55] found that resistance of electroconductive fabrics decreased linearly (from 11,000 Ω to 2000 Ω) with an increase of load up to 110 g, given resistance of around 153 Ω at high weights (>2 kg). The same load (up to 100 g) was applied by Palanisamy et al. [58] on cotton fabrics coated with a polypyrrole conductive polymer, where the resistance was lowered with a higher load, and the warp direction had better fabric sensitivity than the weft.

The change in resistivity for differently treated fabrics was thus measured by the changes in the applied pressure. In this setup, the fabric sample (10 × 10 cm) was placed under the press with insulation material (wood), and pressed from 1 to 10 bar while measuring the conductivity in both weft and warp directions at a distance of 10 cm. As seen from the results presented in Figure 8b, the conductivity of fabrics was generally increasing with the pressure, and the obtained results were again slightly higher in the weft direction when measured on the side containing SWCNTs. For one-layer printing (AP+SWCNT), the conductivity of the back/printed side thus raised from 3∙10^−4^ S/cm up to 8∙10^−4^ S/cm, however, showing a similar trend no matter which side or direction of the fabric was measured. Two-layers back-side printed fabric (PCNF+SWCNT/AP) had almost the same trend as one-layer printed, but generally with the highest conductivity values, up to 1.4∙10^−3^ S/cm. On the other hand, two-side printed samples showed much bigger differences between the measured (back and front) sides, depending on the presence of SWCNTs. The conductivity of the fabric where PCNF+SWCNTs was applied on its front-side, followed by AP on the back-side, was, thus, increasing faster by pressure on the front-side than on the back-side. This effect was even more significant when fabrics were printed with AP on the back-side, with the first layer of PCNF on the front-side, giving the highest conductivities (up to 1.2∙10^−3^ S/cm) on the back- and one of the lowest conductivities on the front-side, being not affected by the increased pressure. The obtained results indicate that SWCNTs form a conductive network independent of how they had been applied, through PCNF or AP. 

Such treatments could be attractive for fabrics being used in flexible and wearable electronics, particularly inexpensive and simple electric circuits [4], textile electrodes [57], sensors and actuators [56], when using higher SWCNTs’ concentrations to raise the conductivity by at least one order. On the other hand, the obtained conductivity ranges of SWCNT-printed fabric sides (10^−4^–10^−3^ S/cm) fell on the edge between conductive and dissipative materials with an antistatic property (<10^−8^), electrostatic discharge (ESD) protection (10^−8^–10^−2^ S/cm) [59,60], or an electromagnetic interference (EMI) shielding effect (10^−2–^10^2^ S/cm) [59], showing applications in various protective clothing and smart textiles (heating, communicating) [61].

### 3.8. Tensile and Tear Strengths, and Breaking Elongation Properties

**Figure 9:**
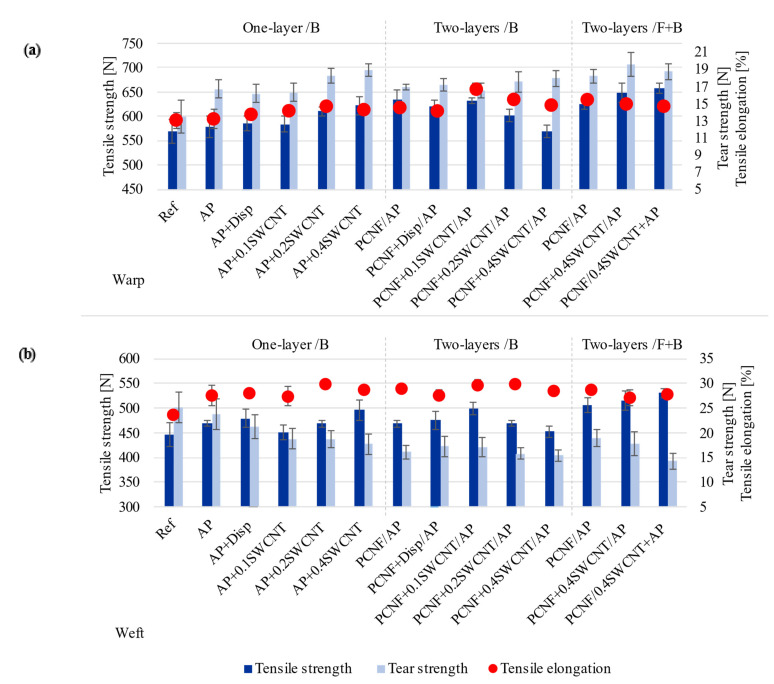
The effect of printing on the fabric’s tensile and tear strength, and elongation, measured in the warp (**a**) and weft (**b**) directions.

The tensile strength and elongation and tear strength properties of untreated and differently printed fabrics, in both warp and weft directions are presented in Figure 9. The fabric had two weft systems and, thus, much higher weft density (51 threads/cm) in comparison with warp density (36 threads/cm). The results also show that printing the fabric with AP improved its tensile strength in both directions (from 569 N to 579 N in the warp direction and from 446 N to 469 N in the weft direction), while pre-printing of PCNF gave an additional effect (to 635 N and 468 N, meaning an 11% and 5% improvement in the warp and weft directions, respectively), which was, for both-sided printing, improved even further in the weft direction (14% increase) and slightly lowered in the warp direction (9%). The addition of SWCNTs to AP increased the tensile strength in both directions around 10% (up to 622 N/warp and 495 N/weft) with the increasing of NPs’ content, which decreased when SWCNTs were printed together with PCNF on the same side. When SWCNTs were applied together with AP in both-sided printing, the highest tensile strengths in both directions were obtained (an increase of around 16%/warp and 19%/weft). The elongation values followed a similar trend, but were much less pronounced in both directions. The results coincided well with the coating’s mass change (Figure 3b), where the higher mass gave higher tensile strength properties. 

The tear strength of fabrics was much lower in the warp direction (around 13.5 N) compared to the weft (25.1 N), due mainly to the fabric’s two-wefts assembly, meaning higher weft threads’ density and, consequently, higher tear resistance. However, an obvious improvement (23.7 %) of warp tear strength and slight reduction (5.5%) of weft tear strength is observed by printing the AP. When PCNF was pre-printed, slightly higher values were obtained in the warp direction (one and both-sided), being even further improved with the addition of SWCNT (one sided—32%/17.9 N and both-sided with SWCNTs applied with PCNF—43%/19.5 N). On the other hand, the tear strength in the weft direction was reduced even up to 44% as compared to the non-treated fabric. Such properties are related to the fabric’s construction, where the AP/PCNF coating affects the predominant weft threads by moving them further apart (Figure 1), thus lowering the tear resistance. In addition, reduced tear strength is common when NPs are coated on the fabric or the coating thickness is high, forming cracks due to internal stress [62]. The SWCNTs’ dispersant did not improve nor destroy the mechanical properties of the fabrics. Our results are comparable to some other researches, for example, Cui et al. [13] improved tensile strength by 20%, while elongation at break decreased by 21% for cotton treated with 12 wt% MWCNTs after the continuous dip-coating method. Liu et al. [16] increased the tensile strength of cotton treated with 0.5–5 wt% of MWCNTs by 4–22% in the warp and 15–51% in the weft direction and tear strength 17–41% in the warp and 2–27% in weft directions.

### 3.9. Abrasion Resistance Properties

**Figure 10:**
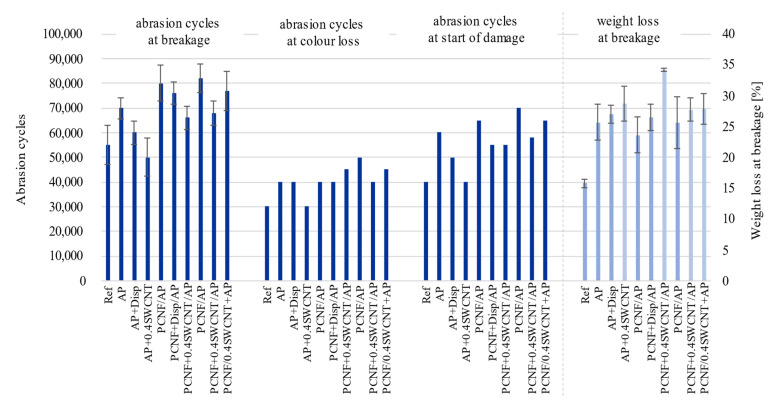
The effect of printing on the fabric’s abrasion resistance and weight loss properties.

Abrasion resistance, together with weight loss and colour loss was assessed on the fabric’s front side (turned towards the skin), where the weight loss and the number of cycles are defined at the fabric’s breakdown (at least two threads need to be torn). Figure 10 shows that AP treatment improved the resistance from 55,000 to 70,000 cycles (compared to non-treated) together with higher weight loss. The pre-printing of PCNF additionally improved the breakage to 80,000 cycles, while the two-sided printing method gave slightly higher abrasion results with 82,000 cycles, which might be due to a better connection between the fibres (SEM, Figure 1), causing better resistance to higher and long-term applied stress. On the other hand, the addition of SWCNTs to AP or PCNF has the opposite effect, independent of the printing strategy, since the breakage already occurred at 50,000 and 66,000 cycles for one-sided printing, and at 77,000 cycles when SWCNTs were applied within AP for a both-sided printing sample. The same trend followed the starting point when damages occurred, which was improved greatly by applying PCNF (from 40,000/AP to 65,000/two-layer printing up to 70,000 cycles/two-sided), and reduced with SWCNTs’ addition. SWCNTs treated samples have around 5% higher weight loss than samples without it, due to the lower abrasion resistance caused by weaker connections between the fibres. Nanoparticles present on fabrics usually decrease the fabric’s resistance to abrasion, since the number of contact points is higher, which, consequently, enlarges the friction force. [62].

## 4. Conclusions

Flame-retardant fabric was screen-printed with phosphorylated cellulose nanofibrils (PCNFs) followed by hydrophobic polyacrylate (AP), with and without the addition of 0.1–0.4 wt% pre-dispersed SWCNTs as one or two layer coatings, and applying the PCNF on the front-side, or together with AP on the back-side to improve its thermal, UV protection and thermophysiological comfort properties (moisture adsorption, water vapour and heat transfer). The results showed that the fabric properties were influenced primarily by the printing strategy and secondly by the amount of coatings applied, where the best results were achieved by both-sided printing where SWCNTs were applied within the AP paste on the back-side, while PCNF was pre-printed on the front-side. Such a treated fabric, apart from having much better UV protective properties while given a water repellent outside-facing side and hydrophilic skin-facing side, also has an improved heat and water-vapour transfer, thermal resistance and tensile strength. In addition, a pressure-sensing electrical conductivity SWCNT-printed side ranks such a fabric among the antistatic, electrostatic discharge (ESD) or electromagnetic interference (EMI) shielding protectives. This work shows the significant potential of using SWCNTs along with nanocellulose as an efficient, user-friendly and comfortable wear alternative to conventional textile finishing.

## References

[1] Song G., Mandal S., Rossi R.M., Song G., Mandal S., Rossi R.M. (2017). 4—Development of high performance thermal protective clothing. Thermal Protective Clothing for Firefighters.

[2] Norouzi M., Zare Y., Kiany P. (2015). Nanoparticles as effective flame retardants for natural and synthetic textile polymers: Application, mechanism, and optimization. Polym. Rev..

[3] Horrocks A.R. (2011). Flame retardant challenges for textiles and fibres: New chemistry versus innovatory solutions. Polym. Degrad. Stab..

[4] Rosace G., Trovato V., Colleoni C., Caldara M., Re V., Brucale M., Piperopoulos E., Mastronardo E., Milone C., de Luca G. (2017). Structural and morphological characterizations of MWCNTs hybrid coating onto cotton fabric as potential humidity and temperature wearable sensor. Sens. Actuators B Chem..

[5] Krucińska I., Skrzetuska E., Urbaniak-Domagala W. (2012). Prototypes of carbon nanotube-based textile sensors manufactured by the screen printing method. Fibres Text. East. Eur..

[6] Grancarić A.M., Jerković I., Koncar V., Cochrane C., Kelly F.M., Soulat D., Legrand X. (2017). Conductive polymers for smart textile applications. J. Ind. Text..

[7] Karim N., Afroj S., Leech D., Abdelkader A.M., Ray A.K. (2021). Flexible and Wearable Graphene-Based E-Textiles. Oxide Electronics.

[8] Knittel D., Schollmeyer E. (2009). Electrically high-conductive textiles. Synth. Met..

[9] Wang Y., Weng G.J., Meguid S.A., Weng G.J. (2018). Electrical Conductivity of Carbon Nanotube- and Graphene-Based Nanocomposites. Micromechanics and Nanomechanics of Composite Solids.

[10] Abbas A., Zhao Y., Zhou J., Wang X., Lin T. (2013). Improving thermal conductivity of cotton fabrics using composite coatings containing graphene, multiwall carbon nanotube or boron nitride fine particles. Fibers Polym..

[11] Mondal S., Hu J. (2007). A novel approach to excellent UV protecting cotton fabric with functionalized MWNT containing water vapor permeable PU coating. J. Appl. Polym. Sci..

[12] Jhon Y.I., Kim C., Seo M., Cho W.J., Lee S., Jhon Y.M. (2016). Tensile characterization of single-walled carbon nanotubes with helical structural defects. Sci. Rep..

[13] Cui J., Zhou S. (2018). Highly conductive and ultra-durable electronic textiles via covalent immobilization of carbon nanomaterials on cotton fabric. J. Mater. Chem. C.

[14] Shahidi S., Moazzenchi B. (2018). Carbon nanotube and its applications in textile industry—A review. J. Text. Inst..

[15] Rahman M.J., Mieno T. (2015). Conductive cotton textile from safely functionalized carbon nanotubes. J. Nanomater..

[16] Liu Y., Tang J., Wang R., Lu H., Li L., Kong Y., Qi K., Xin J.H. (2007). Artificial lotus leaf structures from assembling carbon nanotubes and their applications in hydrophobic textiles. J. Mater. Chem. C.

[17] Liu Y., Wang X., Qi K., Xin J.H. (2008). Functionalization of cotton with carbon nanotubes. J. Mater. Chem. C.

[18] Li G., Wang H., Zheng H., Bai R. (2010). A facile approach for the fabrication of highly stable superhydrophobic cotton fabric with multi-walled carbon nanotubes-azide polymer composites. Langmuir ACS J. Surf. Colloids.

[19] Nafeie N., Montazer M., Nejad N.H., Harifi T. (2016). Electrical conductivity of different carbon nanotubes on wool fabric: An investigation on the effects of different dispersing agents and pretreatments. Colloids Surf. A Physicochem. Eng. Asp..

[20] Lin J.-H., Lin Z.-I., Pan Y.-J., Hsieh C.-T., Lee M.-C., Lou C.-W. (2016). Manufacturing techniques and property evaluations of conductive composite yarns coated with polypropylene and multi-walled carbon nanotubes. Compos. A Appl. Sci. Manuf..

[21] Lin Z.-I., Lou C.-W., Pan Y.-J., Hsieh C.-T., Huang C.-H., Huang C.-L., Chen Y.-S., Lin J.-H. (2017). Conductive fabrics made of polypropylene/multi-walled carbon nanotube coated polyester yarns: Mechanical properties and electromagnetic interference shielding effectiveness. Compos. Sci. Technol..

[22] Xue C.-H., Wu Y., Guo X.-J., Liu B.-Y., Wang H.-D., Jia S.-T. (2020). Superhydrophobic, flame-retardant and conductive cotton fabrics via layer-by-layer assembly of carbon nanotubes for flexible sensing electronics. Cellulose.

[23] Mahmoudifard M., Safi M. (2012). Novel study of carbon nanotubes as UV absorbers for the modification of cotton fabric. J. Text. Inst..

[24] Lee T.-W., Han M., Lee S.-E., Jeong Y.G. (2016). Electrically conductive and strong cellulose-based composite fibers reinforced with multiwalled carbon nanotube containing multiple hydrogen bonding moiety. Compos. Sci. Technol..

[25] Kokol V., Vivod V., Peršin Z., Kamppuri T., Dobnik-Dubrovski P. (2021). Screen-printing of microfibrillated cellulose for an improved moisture management, strength and abrasion resistant properties of flame-resistant fabrics. Cellulose.

[26] El-Shafei A.M., Adel A.M., Ibrahim A.A., Al-Shemy M.T. (2019). Dual functional jute fabric biocomposite with chitosan and phosphorylated nano-cellulose (antimicrobial and thermal stability). Int. J. Biol. Macromol..

[27] Thomas B., Raj M.C., Joy J., Moores A., Drisko G.L., Sanchez C. (2018). Nanocellulose, a versatile green platform: From biosources to materials and their applications. Chem. Rev..

[28] ISO (1977). Textiles—Woven Fabrics—Determination of Mass per Unit Length and Mass per Unit Area.

[29] ISO (1996). Textiles—Determination of Thickness of Textiles and Textile Products.

[30] CEN (1993). Textiles—Woven Fabrics—Construction—Methods of Analysis—Part 2: Determination of Number of Threads per Unit Length.

[31] ISO (2012). Textiles—Domestic Washing and Drying Procedures for Textile Testing.

[32] ISO (2005). Textiles—Standard Atmospheres for Conditioning and Testing.

[33] ISO (1995). Textiles—Determination of the Permeability of Fabrics to Air.

[34] ASTM (2008). Surface Wettability and Absorbency of Sheeted Materials Using an Automated Contact Angle Tester.

[35] ISO (2014). Textiles—Physiological Effects—Measurement of Thermal and Water-Vapour Resistance under Steady-State Conditions (Sweating Guarded-Hotplate Test).

[36] ISO (2016). Protective Clothing—Protection against Flame—Method of Test for Limited Flame Spread.

[37] CEN (2001). Textiles—Solar UV Protective Properties—Part 1: Method of Test for Apparel Fabrics.

[38] ISO (2013). Textiles—Tensile Properties of Fabrics—Part 1: Determination of Maximum Force and Elongation at Maximum Force Using the Strip Method.

[39] ISO (2000). Textiles—Tear Properties of Fabrics—Part 2: Determination of Tear Force of Trouser-Shaped Test Specimens (Single Tear Method).

[40] ISO (2016). Textiles—Determination of the Abrasion Resistance of Fabrics by the Martindale Method—Part 2: Determination of Specimen Breakdown.

[41] ISO (1998). Textiles—Determination of the Abrasion Resistance of Fabrics by the Martindale Method—Part 3: Determination of Mass Loss.

[42] ISO (1998). Textiles—Determination of the Abrasion Resistance of Fabrics by the Martindale Method—Part 4: Assessment of Appearance Change.

[43] Huang J. (2006). Thermal parameters for assessing thermal properties of clothing. J. Therm. Biol..

[44] El Messiry M., El Ouffy A., Issa M. (2015). Microcellulose particles for surface modification to enhance moisture management properties of polyester, and polyester/cotton blend fabrics. Alex. Eng. J..

[45] Kwon S.-o., Kim J., Moon M.-W., Park C.H. (2016). Nanostructured superhydrophobic lyocell fabrics with asymmetric moisture absorbency: Moisture managing properties. Text. Res. J..

[46] Havenith G., Elsner P., Hatch K., Wigger-Alberti W. (2003). Clothing and Thermoregulation. Textiles and Skin.

[47] Ashraf M., Siyal M., Nazir A., Rehman A. (2016). Single-step antimicrobial and moisture management finishing of pc fabric using zno nanoparticles. Autex Res. J..

[48] Das B., Das A., Kothari V., Fanguiero R., Araújo M. (2007). Moisture transmission through textiles. Part I: Processes involved in moisture transmission and the factors at play. Autex Res. J..

[49] Kwon S.-o., Ko T.-J., Yu E., Kim J., Moon M.-W., Park C.H. (2014). Nanostructured self-cleaning lyocell fabrics with asymmetric wettability and moisture absorbency (Part I). RSC Adv..

[50] Noman M., Petru M., Amor N., Louda P. (2020). Thermophysiological comfort of zinc oxide nanoparticles coated woven fabrics. Sci. Rep..

[51] Wang T., Xu X., Ren Y., Qin S., Sui X., Wang L. (2014). Kinetics of thermal degradation of viscose fiber and fire retardant viscose fiber. J. Eng. Fibers Fabr..

[52] Linda A., Demšar A., Varga K. (2016). Termična Analiza Ognjevarnih Vlaken: Diplomsko Delo. Bachelor‘s Thesis.

[53] Horrocks A.R., Smith W.C. (2019). 9—Smart Flame Retardant Textile Coatings and Laminates. Smart Textile Coatings and Laminates.

[54] Tokarska M., Frydrysiak M., Zięba J. (2013). Electrical properties of flat textile material as inhomegeneous and anisotropic structure. J. Mater. Sci. Mater. Electron..

[55] Tseghai G.B., Malengier B., Nigusse A., Van Langenhove L. (2018). Development and Evaluation of Resistive Pressure Sensors from Electro-Conductive Textile Fabric. Proceedings of the The Second International Forum on Textiles for Graduate Students (IFTGS).

[56] Giovanelli D., Farella E. (2016). Force sensing resistor and evaluation of technology for wearable body pressure sensing. J. Sens..

[57] Euler L., Guo L., Persson N.-K. (2021). Textile electrodes: Influence of knitting construction and pressure on the contact impedance. Sensors.

[58] Palanisamy S., Tunáková V., Malengier B., Karthik D., Langenhove L., Militký J. (2018). Development of the Force Sensitive Resistors Using Polypyrrole Coated Cotton Woven Fabric for Pressure Sensing Application. Proceedings of the 22nd International Conference STRUTEX, 4 December 2018.

[59] Krifa M. (2021). Electrically conductive textile materials—Application in flexible sensors and antennas. Textiles.

[60] Tokarska M. (2019). Characterization of electro-conductive textile materials by its biaxial anisotropy coefficient and resistivity. J. Mater. Sci. Mater. Electron..

[61] Hebeish A.A., El-Gamal M.A., Said T.S., El-Hady R.A.M.A. (2010). Major factors affecting the performance of ESD-protective fabrics. J. Text. Inst..

[62] Riaz S., Ashraf M., Hussain T., Hussain M.T., Rehman A., Javid A., Iqbal K., Basit A., Aziz H. (2018). Functional finishing and coloration of textiles with nanomaterials. Color. Technol..

